# Theoretical study on aromatic and open-shell characteristics of carbon nanobelts composed of indeno[1,2-*b*]fluorene units: dependence on the number of units and charge states†

**DOI:** 10.1039/d0ra04787b

**Published:** 2020-07-07

**Authors:** Ryohei Kishi, Masaki Yamane, Ryosuke Sugiura, Wataru Yoshida, Yosuke Shimizu, Masayoshi Nakano

**Affiliations:** Department of Materials Engineering Science, Graduate School of Engineering Science, Osaka University 1-3 Machikaneyama Toyonaka Osaka 560-8531 Japan mnaka@cheng.es.osaka-u.ac.jp; Center for Quantum Information and Quantum Biology (QIQB), Institute for Open and Transdisciplinary Research Initiatives, Osaka University 1-3 Machikaneyama Toyonaka Osaka 560-8531 Japan; Center for Spintronics Research Network (CSRN), Graduate School of Engineering Science, Osaka University 1-3 Machikaneyama Toyonaka Osaka 560-8531 Japan

## Abstract

In this study, we theoretically investigate the aromatic and open-shell characteristics of carbon nanobelts (CNBs) composed of five- and six-membered rings. We have designed nanobelts composed of indeno[1,2-*b*]fluorene ([1,2-*b*]IF) units, which are referred to as [*N*]IF-CNB (*N*: the number of five-membered rings). The number of π-electrons, *n*_π_, in neutral [*N*]IF-CNB is 7*N*, and thus depending on *N* and charge states, *n*_π_ can be 4*n* + 2 and 4*n*. Quantum chemical calculations on neutral [6]IF-CNB and [8]IF-CNB and dicationic [8]IF-CNB^2+^ have revealed that they are expected to exhibit unique aromatic and open-shell characteristics depending on *n*_π_, there are several analogies of the electronic structures in [*N*]IF-CNB to those in [*N*]annulene. Delocalized and intermediate open-shell electronic structures of [*N*]IF-CNB are also useful to drastically change the third-order nonlinear optical properties. These results suggest that theoretically designed [*N*]IF-CNB can be attractive and challenging targets of organic synthesis for realizing novel open-shell functional conjugated macrocycles.

## Abstract 2

Dependence of aromatic and open-shell characteristics on the number of units and charged states was theoretically investigated for carbon nanobelts composed of indeno[1,2-*b*]fluorene units by using quantum chemical calculations.

## Graphical abstract

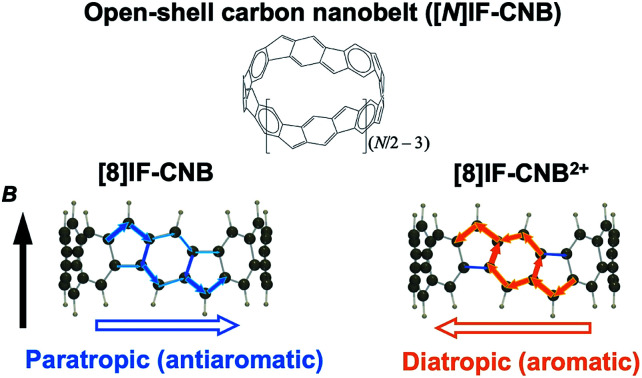

## Introduction

Creation of π-conjugated oligomers with well-defined cyclic structures, *i.e.*, conjugated macrocycles, has been one of the hot topics in materials science.^1^ Owing to the cyclic topologies, the conjugation lengths of fully conjugated macrocycles are regarded as infinite, and this feature is reflected in their unique delocalized electronic structures. Such electronic structures are closely related to their geometries, stabilities, properties and related functionalities. Recent progress in experimental techniques have allowed us to realize a variety of conjugated macrocycles.^1–11^ One of the most important features of conjugated macrocycles is that there are inner and outer domains that are spatially separated from one another by the cyclic backbone. Since conjugated macrocycles can offer different electrostatic environments in the inner and outer domains, they are also considered to be useful as efficient materials for molecular recognition and host–guest chemistry.^11–17^ Not only planar, but also non-planar conjugated macrocycles with curved π-conjugation surface, such as Möbius-, saddle- and belt-shaped systems, are very interesting materials in this regard.^11,18^ Even though synthesis of such non-planar conjugated macrocycles with well-defined curved structures is one of the most challenging topics in synthetic chemistry, there have been plenty of successes to realize such materials, including cycloparaphenylenes (CPPs), extended porphyrinoids and carbon nanobelts (CNBs).^1–19^

Aromaticity is an important chemical concept for characterizing the delocalized electronic structures of π-conjugated systems involving π-conjugation rings. According to Hückel rule for a planar π-conjugation ring, the number of π-electrons, *n*_π_, can be the simplest index for characterizing its aromaticity.^20^ Aromatic compounds with (4*n* + 2)π electrons are known to exhibit characteristic thermodynamic stabilities and properties, whereas antiaromatic compounds with 4*n*π electrons tend to be rather unstable. The concept of aromaticity and the classification of compounds by *n*_π_ are extensively used to characterize the electronic structures of various π-conjugated systems.^21^

Since the aromaticity concept is closely related to the electron occupancies and degeneracy of the frontier molecular orbitals (MOs), it is also connected to another important chemical concept concerning the electron occupancies and degeneracy, *i.e.*, open-shell character.^22,23^ For example, antiaromatic 4*n*π-electron systems tend to have very small energy gap between the highest occupied and lowest unoccupied MOs (HOMO and LUMO). When the HOMO and LUMO characterize bonding and anti-bonding natures for a specific or effective chemical bond, such a system tends to have open-shell (diradicaloid) electronic structure in the singlet state.^23^ Indeed, most antiaromatic compounds are known to be highly reactive, since the electronic structures of open-shell systems are easily fluctuated by external physical and chemical perturbations. Such a feature of open-shell electronic structure is, on the other hand, expected to be useful for creation of novel stimuli-responsive functional materials, such as efficient nonlinear optical (NLO) materials,^24^ if one can increase their thermodynamic and kinetic stabilities by molecular and materials design strategies.^23^ In this regard, a theoretical index, diradical character *y* [0 (closed-shell) ≤ *y* ≤ 1 (pure diradical)],^25^ which characterizes the degree of open-shell of an electron pair, has been shown to be a useful index to design novel open-shell functional molecules with unique electronic, optical and magnetic response properties.^24^

**Fig. 1:**
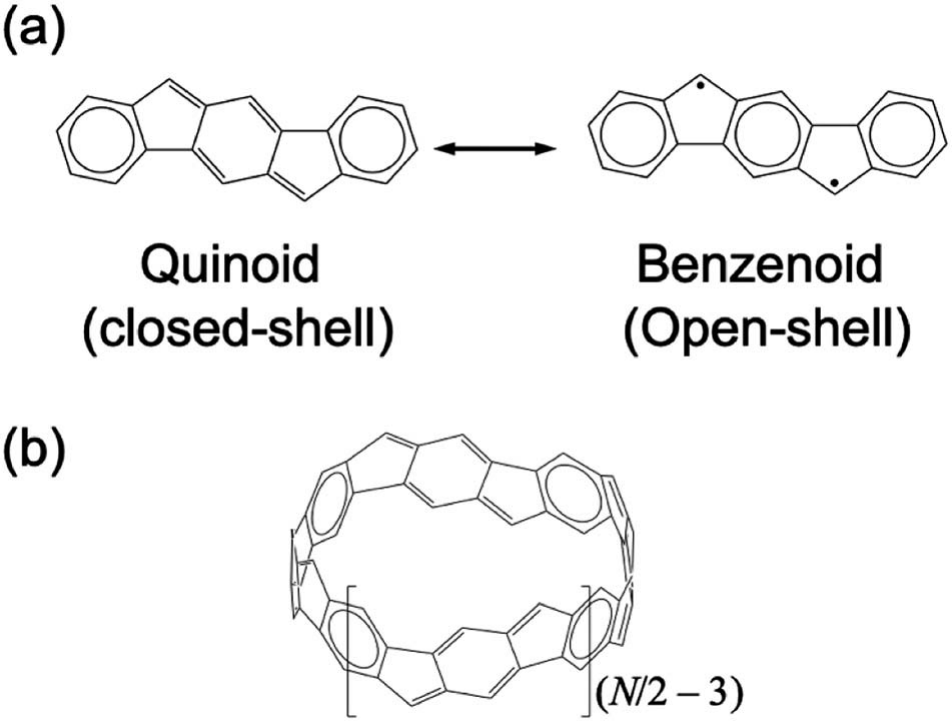
Quinoid (closed-shell)/benzenoid (open-shell) resonance structure of indeno[1,2-*b*]fluorene (a), and structure of [*N*]IF-CNB (*N* ≥ 6) (b). Definition of coordinate axis is explained in the computational details.

In order to realize novel open-shell functional molecules, it is very important to construct practical design strategies for tuning *y* by chemical modifications, such as inclusion of *para*-quinodimethane (*p*QM) substructures into the molecular skeleton in order to tune quinoid (closed-shell)/benzenoid (open-shell) resonance structure.^26^ For example, several fused polycyclic hydrocarbons involving plural five-membered rings can involve *p*QM substructures, and thus there are several real open-shell molecules with a variety of *y* values in this class. These systems are also known to exhibit a variety of antiaromatic characters. Indenofluorene (IF), where five- and six-membered rings are alternately fused, is one of the typical antiaromatic open-shell molecules (for possible resonance structure, see Fig. 1a).^27^ Depending on the topologies of ring-fusion, the regioisomers of IF are shown to exhibit different *y* values, and thus, several derivatives of IF are expected to be useful building units for creation of open-shell macromolecules.^27^ Indeed, Nobusue, Shimizu and Tobe reported synthesis of planar tetracyclopenta[*def*,*jkl*,*pqr*,*vwx*]tetraphenylene (TCPTP, 28π electrons), which involves repeating indeno[2,1-*c*]fluorene substructures.^28^ Quantum chemical calculations combined with the experimental analysis revealed that the *D*_4h_ structure having almost pure diradical and weak tetraradical characters is considered to be a transition state between the almost closed-shell *D*_2h_ local minima.^28^ On the other hand, within the planar fully-fused conjugated macrocycles, increasing the number of repeating IF substructures is considered to be difficult because of the structural limitations.

In our previous study, we have theoretically investigated the relationship between the geometric features, electronic structures and third-order NLO properties of a non-planar conjugated macrocycle, *i.e.*, a carbon nanobelt‡‡According to the ref. 19, the term “carbon nanobelt” is used for a class of belt-shaped hydrocarbons composed only of six-membered rings.^19^ In this classification, belt-shaped compounds involving five-membered rings are classified into “aromatic belt”. However, we here call the present belt-shaped hydrocarbons involving five-membered rings, [*N*]IF-CNB (*i.e.*, IF-containing carbon nanobelt) as a counterpart of regular CNB, because they can exhibit both aromatic and antiaromatic characters depending on *N*, and the term “aromatic belt” may cause confusion for readers about the aromatic characters of [*N*]IF-CNB. composed of five- and six-membered rings. Here we call the system [6]IF-CNB (Fig. 1, *N* = 6), where *N* corresponds to the number of five-membered rings involved in [*N*]IF-CNB.^29^ The molecular design of [6]IF-CNB was inspired from those of indeno[1,2-*b*]fluorene and (6,6)CNB, the latter of which has been synthesized by Itami and coworkers.^19^ [*N*]IF-CNB involves repeating indeno[1,2-*b*]fluorene substructures (see Fig. 1a), and owing to its belt-shape, we can reasonably increase the number of units without changing the repeating structures. Note that only even numbers of *N* are allowed in [*N*]IF-CNB to make a reasonable cyclic structure of hoop.

One of interesting features of [*N*]IF-CNB is the number of π-electrons, *n*_π_ = 7*N*. In other words, *n*_π_ = 4*n* + 2 when *N* = 4*m* + 2 (*m* = 1, 2, …), while *n*_π_ = 4*n* when *N* = 4*m*, and thus unique aromatic characters are expected if they are fully π-conjugated. Actually, [6]IF-CNB is expected to exhibit weak global aromatic character from the analysis of the magnetically induced ring-current (MIC). Analysis of the MO levels of [6]IF-CNB has revealed that there is an analogy in the orbital degeneracy of [6]IF-CNB to that of [6]annulene. Furthermore, [6]IF-CNB was shown to exhibit an intermediate multiradical character owing to the presence of IF substructures, the feature of which is reflected in the enhancement of third-order NLO property of [6]IF-CNB compared to that of closed-shell (6,6)CNB. Since [*N*]annulene is known to drastically change their structures, stabilities and properties by changing the number of π-electrons, the results of [6]IF-CNB have stimulated us to further investigate *n*_π_-dependence of electronic structures and physico-chemical properties of [*N*]IF-CNB. In this study, we therefore conduct several theoretical analyses in order to clarify the relationship between *n*_π_ (depending on the number of units and charge states), aromatic and open-shell characters, and NLO properties of [*N*]IF-CNB. On the basis of the results, we will discuss a novel design strategy for creation of open-shell functional materials. In order to focus on the fundamental relationship between *n*_π_ and electronic structures (aromatic and open-shell characters), we here discuss the results of neutral [6]IF-CNB (*n*_π_ = 42; [4*n* + 2]π), [8]IF-CNB (*n*_π_ = 56; 4*n*π) and dicationic [8]IF-CNB^2+^(*n*_π_ = 54; [4*n* + 2]π).

## Computational details

All the carbon nanobelts investigated in this study are expected to have open-shell characters in the singlet state. For such systems, spin-unrestricted density functional theory (UDFT) are known to sometimes give incorrect bond-length alternation (BLA) patterns around the radical sites.^30^ Geometry optimizations followed by frequency analysis were therefore performed at the RB3LYP/6-311G* level of approximation, which is expected to reproduce BLA patterns for several intermediate multiradicaloids involving five-membered rings.^30^ In this study, because of the unique cyclic structure of [*N*]IF-CNB, we have carefully discussed DFT functional dependence of the optimized geometry for [*N*]IF-CNB. For details, please see the ESI.† Diradical character for the *i*-th natural orbital (NO) pair (*y*_*i*_) was evaluated from the occupation number of the *i*-th lowest-unoccupied NO (LUNO + *i*). Since *y*_*i*_ represents the degree of diradical for a pair of two electrons in the *i*th orbital, a set of *y*_*i*_ is a measure of multiradical characters of the system.^31,32^ Spatial distribution of unpaired electrons can be visualized from the map of odd electron density distribution 
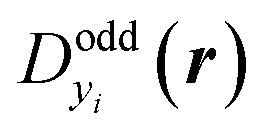
 for *y*_*i*_, which is defined by the spatial distributions of the HONO−*i* and LUNO+*i*, respectively) {*ϕ*_HONO−*i*_(***r***) and *ϕ*_LUNO+*i*_(***r***)} and *n*_LUNO+*i*_ as,^32^
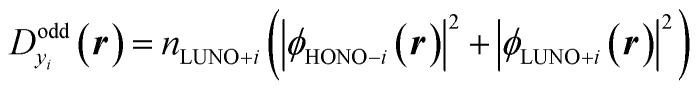
where the spatial integration of 
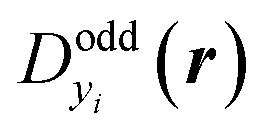
 is related to *y*_*i*_ by the following equation,
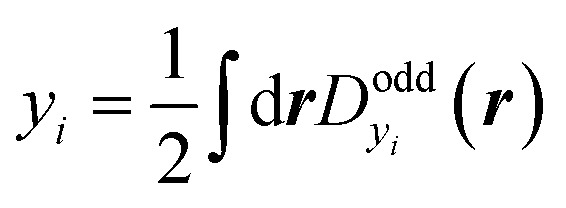


If *y*_*i*_ and *y*_*i*+1_ are the same, the corresponding NOs for *y*_*i*_ and *y*_*i*+1_ are degenerate, so that these NOs are not necessarily the symmetry-adapted ones. We therefore need to take the sum or the arithmetic average of 
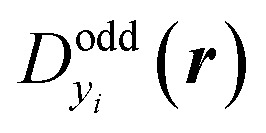
 and 
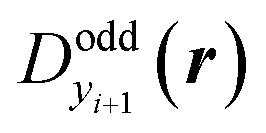
 to illustrate the symmetry-adapted odd-electron densities.^29^ The result of *y*_*i*_ is known to depend on the employed quantum chemical calculation method.^24^ In this study, we employed a spin-unrestricted long-range corrected exchange-correlation functional, LC-UBLYP,^33^ using the range-separating parameter of *μ* = 0.33 bohr^−1^ along with the 6-31G* basis sets for the calculation and analysis of diradical characters.

There are several criteria to evaluate aromatic characteristics of a system. In order to investigate the aromaticity of the belts, we employed gauge-including magnetically induced current (GIMIC) method to evaluate magnetically induced current (MIC) density.^34^ The unperturbed and magnetically-perturbed electron densities which are needed for GIMIC calculations were evaluated with the LC-UBLYP exchange-correlation functional with a range separating parameter *μ* = 0.33 bohr^−1^ and the 6-311+G** basis set. This functional was found to reproduce the excitation energies, NLO properties such as the second hyperpolarizability (*γ*), and magnetic shielding tensors of a variety of open-shell singlet π-conjugated systems either calculated at higher levels of theory or determined by experiments.^35^ The external magnetic field was applied to the direction going through the hoop (*z* direction). The gauge-invariant atomic orbital (GIAO) method was used throughout the calculation.^36^ Then, the MIC densities was evaluated by GIMIC program. MIC density vector is evaluated at each position of space. In order to clarify the contribution of induced current for each bond, we evaluated bond-integrated MIC density. Here, we set a bisector plane for two atoms (width × depth = 2 Å × 10 Å) so that one of the edges of the plane involves the center of the belt.

The static diagonal second hyperpolarizability tensor *γ*_*iiii*_ which characterizes third-order NLO property at the molecular scale was calculated by using the finite field (FF) approach^37^ combined with the Romberg procedure.^38^ In the Romberg procedure, we employed the same procedure as the previous study.^29^ For all the calculations, the 6-31+G* basis set was adopted. The automatic Romberg differentiations were performed using the T-REX program.^38^ We employed the *B*-convention for the definition of *γ*.^39^ The total energies are evaluated by using the LC-UBLYP (*μ* = 0.33 bohr^−1^) functional with the 6-31+G* basis set. We set the molecular coordinate axis so that *z*-axis is going through the hoop of the belt, which corresponds to the short-axis direction of the belt.^29^ Then, *x*-axis was set to be parallel to the line going through the centers of two face-to-face six-membered rings on the opposite side of the belt.

In order to clarify the spatial contribution of electrons to the second hyperpolarizability tensor *γ*_*iiii*_, we performed the *γ* density analysis.^40^ The *γ* density *ρ*^(3)^_*iii*_(***r***) is defined as the third-order derivative of the electron density *ρ*(***r***) with respect to the applied electric field *F*^*i*^:^40^
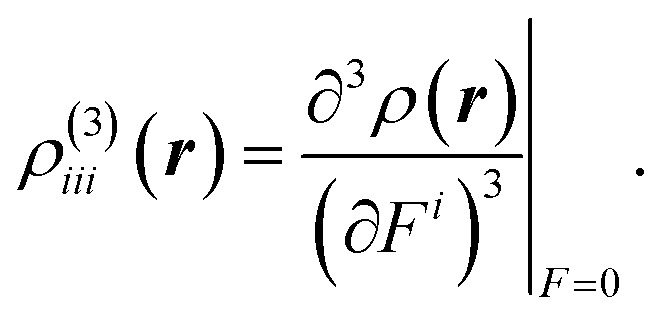


The *γ* density is related to the *γ* value by the following equation,
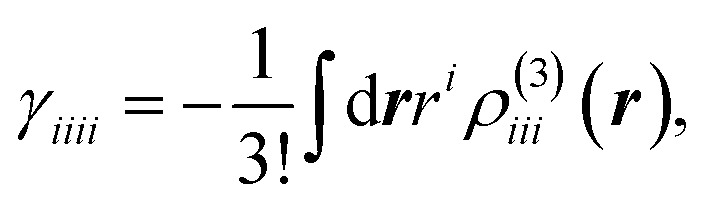
where *r*^*i*^ is the *i*-axis component of the electron coordinate. The *γ* density represents the field-induced third-order response of electron density at position ***r***. A pair of positive and negative *γ* densities contribute to *γ* value, the sign of which is positive (negative) when the direction of vector drawing from positive to negative densities coincides with the positive direction of coordinate axis, and the amplitude of which is proportional to the distance between the positive and negative densities.^40^ All the calculations were carried out using the Gaussian 09 package.^41^ DrawMol package^42^ was used for the visualizations of molecular modelling and spatial distributions of MOs, MIC and *γ* densities.

## Results and discussion

At first, we discuss the local minimum structures of calculated systems. In the previous study, we found that the local minimum structure of neutral [6]IF-CNB belongs to the highest possible *D*_3d_ symmetry. In this study, we have found two local minima with no imaginary frequencies for neutral [8]IF-CNB that belong to the *C*_4v_ and *D*_4_ symmetries, respectively. Energetically more stable (about 7 kcal mol^−1^) structure is found to be the local minimum with the lower *D*_4_ symmetry. The symmetry reduction of the most stable local minimum structure may be similar to the case in TCPTP to some extent, where *D*_4h_ structure is considered to be a transition state between the almost stable *D*_2h_ local minima.^28^ In the case of dicationic [8]IF-CNB^2+^, we found one local minimum structure which belongs to the highest possible *D*_4d_ symmetry. Reduction of symmetries in [8]IF-CNB and recovery of symmetry in dicationic [8]IF-CNB^2+^ are considered to be related to *n*_π_.

**Fig. 2:**
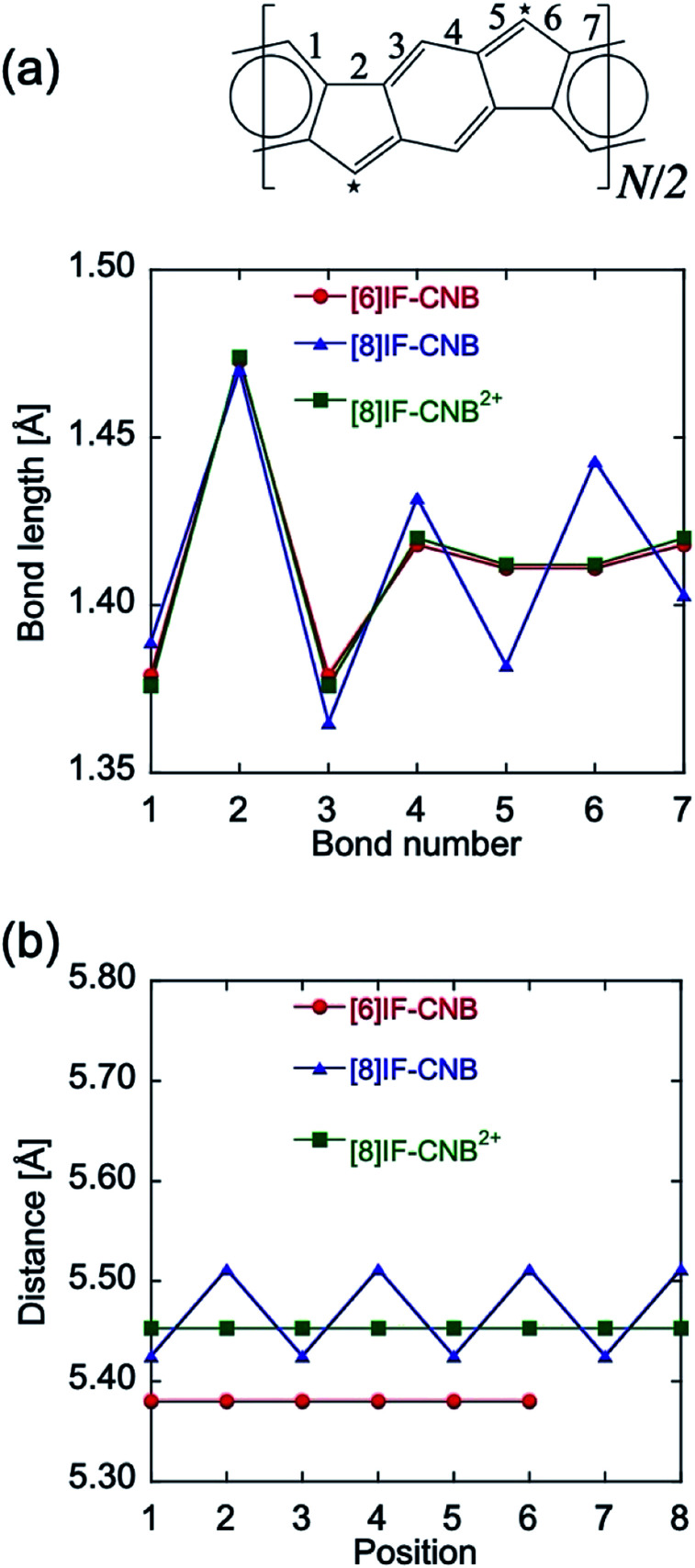
Comparison of bond lengths of selected CC bonds (a), and of distances between the vertices of adjacent five-membered rings (b). For (b), the numbering of the position is arbitrary because of the high symmetry of each system.

Fig. 2a shows the optimized bond lengths of selected CC bonds for the most stable local minimum structure of each system. Only the results of symmetrically unique bonds are given in Fig. 2a. One of the important points is the alternation pattern around the bonds 5 and 6 that belong to the five-membered ring, since it reflects the tendency of quinoid/benzenoid resonance of *p*QM substructures involved in the belt. [6]IF-CNB and [8]IF-CNB^2+^ are shown to exhibit BLA-less features for bonds 5 and 6, whereas [8]IF-CNB is found to exhibit a BLA pattern for these bonds. When we focus on the BLA patterns in the six-membered ring parts, bonds 1 *vs.* 3 (4 *vs.* 7) are shown to be equivalent in [6]IF-CNB and [8]IF-CNB^2+^, and thus, all the six-membered rings involved in the belt are considered to be equivalent in the sense of the resonance structure. On the other hand, these CC bonds are not equivalent in [8]IF-CNB, which means that not all the six-membered rings are equivalent. Judging from the bond lengths of 1 and 7, which are closer to 1.4 Å than those of 3 and 4, there are rather quinoid- and rather benzenoid-like six-membered rings in [8]IF-CNB, and these two-types of six-membered rings appear alternately in the belt.

In order to further examine the quinoid- and benzenoid-like characters of the geometries, we also examined distances between the vertices of adjacent five-membered rings (marked with stars in Fig. 2a). If there is a quinoid-like structure, the corresponding distance between the vertices is expected to become shorter than that for the benzenoid-like structure, *i.e.*, we discuss the alternation patterns of this distance (see Fig. 2b). From the calculation results, averages of the distances are found to increase about 0.1 Å when we increase the number of repeating units *N* from 6 to 8. [8]IF-CNB is shown to exhibit apparent alternations of the distances with a difference of about 0.1 Å, whereas there are no alternation patterns in the cases of [6]IF-CNB and [8]IF-CNB^2+^. The average of the distances is expected to converge to a value for *N* → ∞, which corresponds to the situation of one-dimentional polymer of repeating indeno[1,2-*b*]fluorenes, even though we did not confirm here whether the alternation patterns remains for *N* → ∞ or not.

**Fig. 3:**
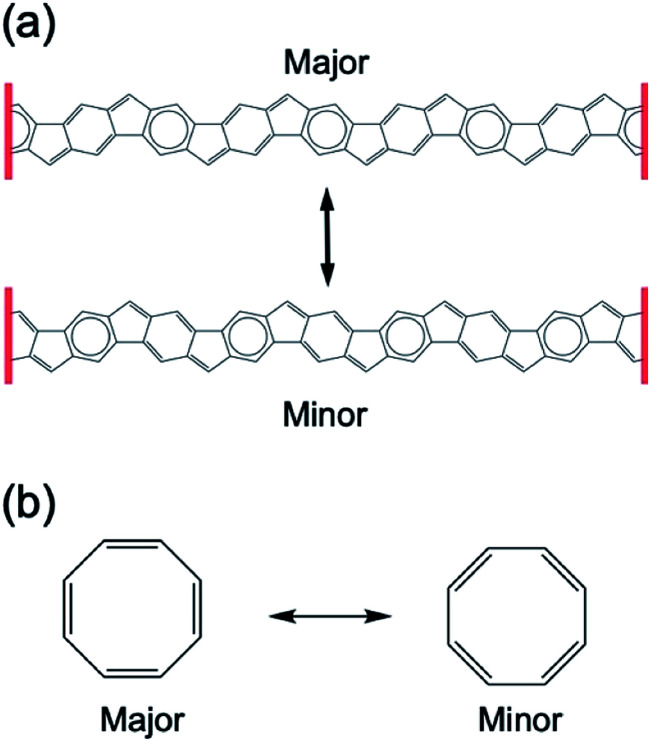
Closed-shell contributions of the resonance structure of [8]IF-CNB (a), and the resonance structure of COT with BLA (b). The major and minor contributions of the resonance forms appear in case of lower symmetric structures.

Judging from these geometric features, we suggest that closed-shell contributions of resonance structures in [8]IF-CNB can be illustrated like that given in Fig. 3a. In the previous study, we discussed the analogy of the resonance structures between [6]IF-CNB and [6]annulene (benzene).^29^ If all the six-membered rings are equivalent, like those in [6]IF-CNB, the contributions of upper and lower forms should be equivalent to each other. However, in [8]IF-CNB (*D*_4_), rather quinoid-like and benzenoid-like structures appear alternately, and thus the weights of these forms in the resonance structure are not considered to be equivalent. Again, we focus on the analogy of the resonance structures between [8]IF-CNB and [8]annulene (cyclooctatetraene, COT). In COT, local minimum structure is non-planar tub-shaped structure that belong to *D*_2d_ symmetry. From the quantum chemical calculations, two types of planar stationary point structures on the potential energy surface (PES) of COT have been predicted. The one belongs to the highest possible *D*_8h_ symmetry with no BLA, and the other does to the *D*_4h_ symmetry with BLA, both of which correspond to the saddle-points on the PES.^43^ For *D*_8h_ structure, two resonance forms shown in Fig. 3b should be equivalent to each other, while for *D*_4h_ or *D*_2d_ structures, the weights of these two forms are not equivalent, depending on how the structure is distorted. Because of the flexible framework of COT, local minimum structure becomes non-planar, and π-electrons are relatively localized on each double bond. On the other hand, [8]IF-CNB is considered to have more rigid framework than COT owing to the fused-ring structure of the belt, and thus significant structural relaxation effect (like that in tub-shaped COT) is expected to be supressed to some extent. From this viewpoint, even though the weights of two forms in the resonance structure given in Fig. 3a are not equivalent, the contribution of minor one may not be negligible. In such case, we can expect delocalized nature of π-electrons even in [8]IF-CNB, which would be reflected in the aromatic character of this system. On the other hand, [6]IF-CNB was shown to have intermediate multiradical characters in our previous theoretical study. We therefore need to investigate the open-shell characters of [8]IF-CNB and [8]IF-CNB^2+^ and the corresponding orbital energy levels, in order to discuss their electronic structures.

**Fig. 4:**
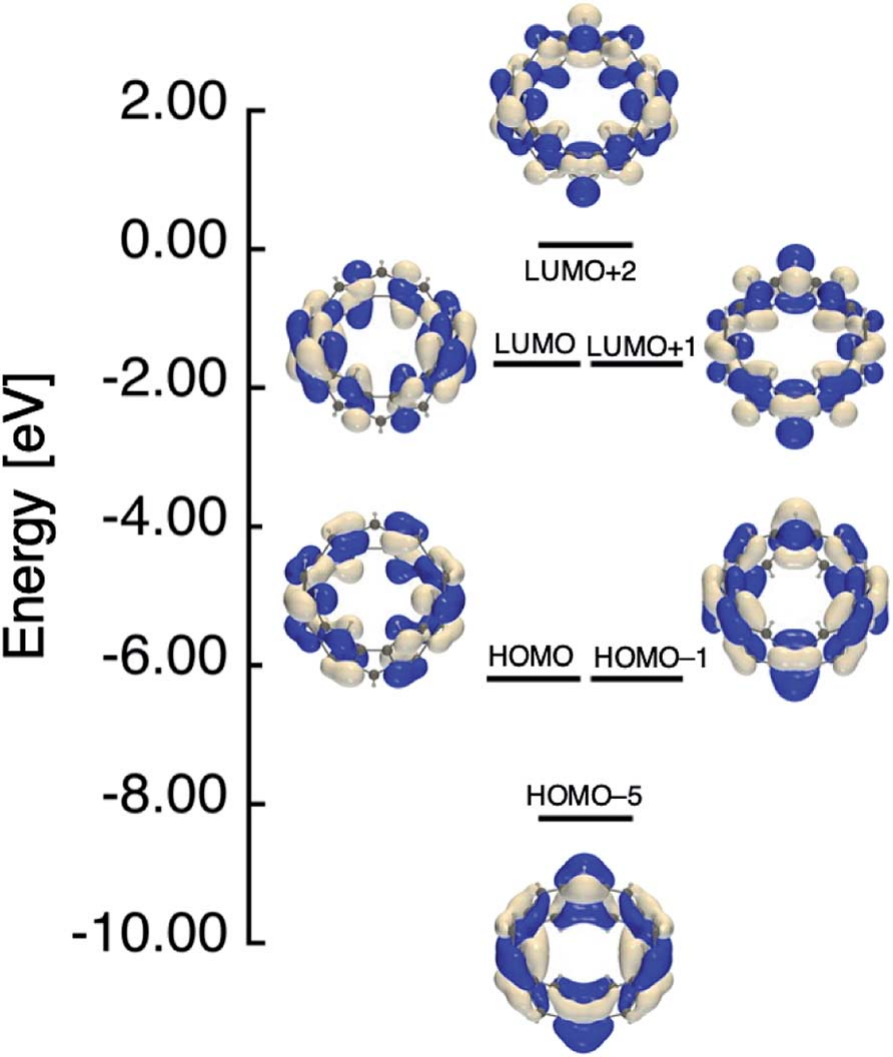
Frontier MO levels of [6]IF-CNB that are related to its aromatic and open-shell characters at the spin-restricted LC-RBLYP/6-31G* level.

**Fig. 5:**
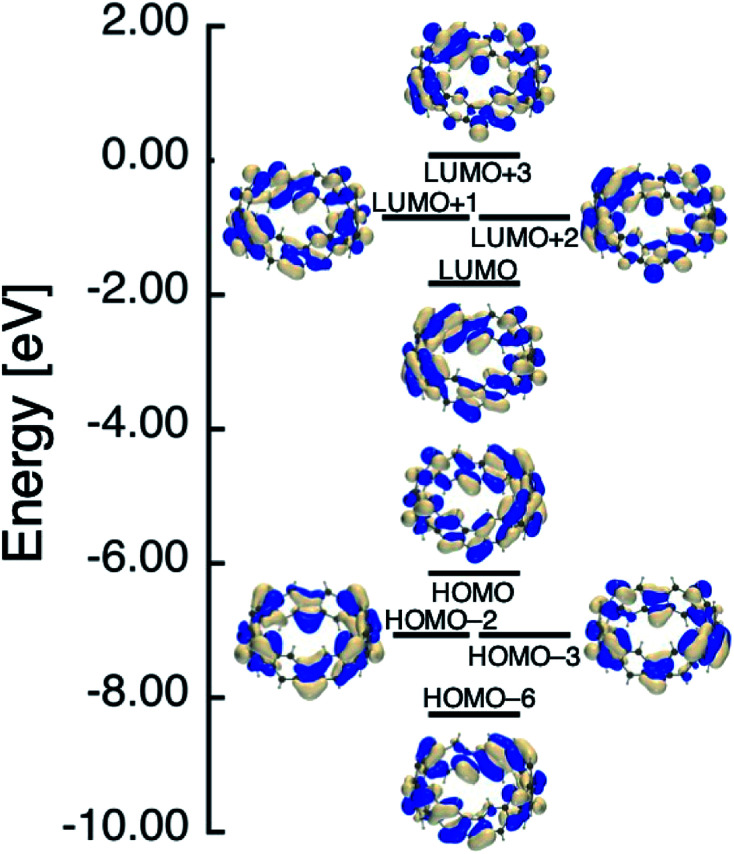
Frontier MO levels of [8]IF-CNB that are related to its aromatic and open-shell characters at the spin-restricted LC-RBLYP/6-31G* level.

**Fig. 6:**
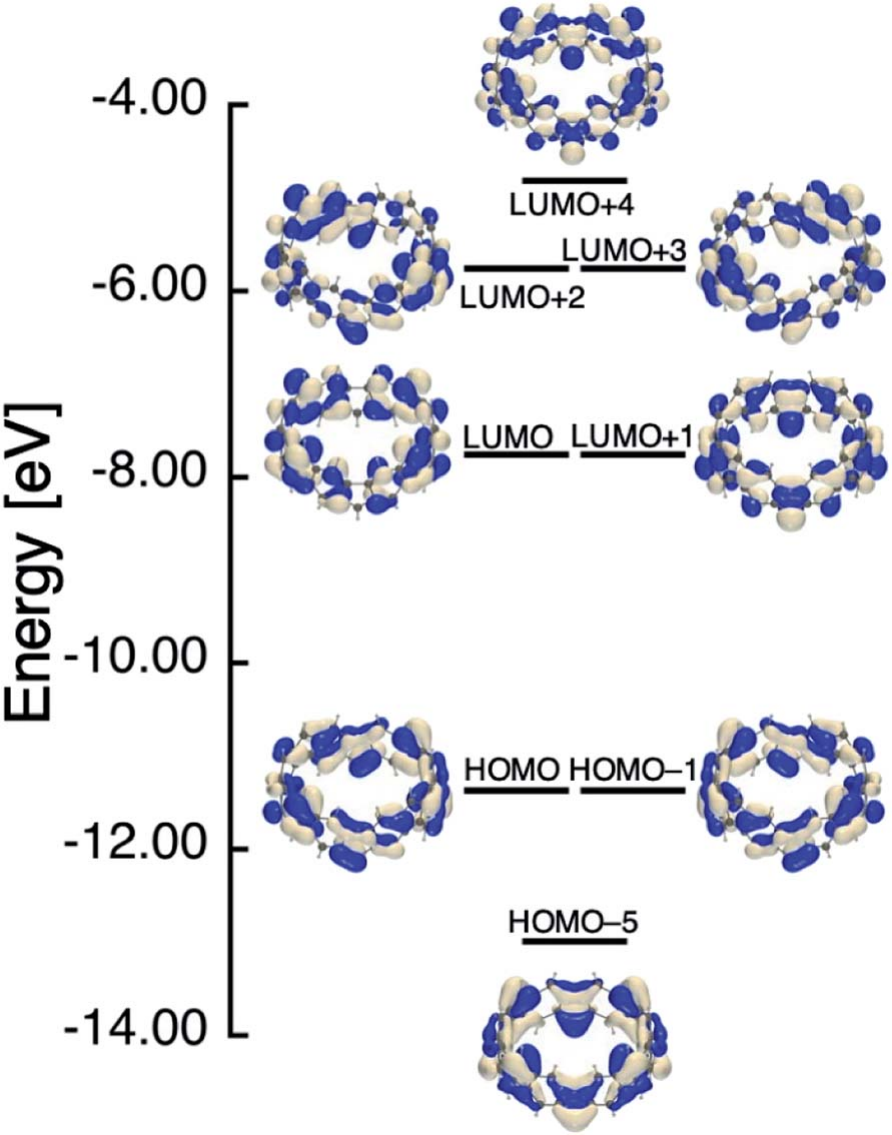
Frontier MO levels of [8]IF-CNB^2+^ that are related to its aromatic and open-shell characters at the spin-restricted LC-RBLYP/6-31G* level.

In Table 1, we summarized calculation results of diradical characters (*y*_*i*_; *i* = 0, 1 and 2). In the previous paper, we found that *y*_0_ and *y*_1_ for [6]IF-CNB become the same value owing to the degenerate nature of the corresponding HOMO/HOMO−1 and LUMO/LUMO+1 levels (see also Fig. 4).^29^ In Fig. 4–6, we therefore summarized the frontier MO levels of these systems at the spin-restricted LC-RBLYP/6-31G* level, where MOs are chosen from the viewpoints of bonding and anti-bonding pairs corresponding to *y*_*i*_, and of the aromatic characters. The HOMO–LUMO gaps obtained at the present level of approximation are considered to be larger than experimental HOMO–LUMO gaps of related open-shell compounds. We employed the LC-RBLYP/6-31G* in consistent to the level for evaluation of *y*_*i*_ (LC-UBLYP/6-31G*). [8]IF-CNB is found to have intermediate *y*_0_ value (0.526), which is larger than *y*_0_/*y*_1_ values of [6]IF-CNB (0.398). In contrast to the results of [6]IF-CNB, the *y*_1_ and *y*_2_ values (0.210) are found to be the same in [8]IF-CNB. Indeed, the MO levels related to these diradical characters, HOMO-2/HOMO-3 and LUMO+1/LUMO+2, are shown to degenerate, respectively (see Fig. 5).

**Table 1:** Summary of calculation results of diradical characters *y*_*i*_ [−] and *γ*_*xxxx*_ [10^3^ a.u.] for the obtained local minimum structures

System	*y*_0_	*y*_1_	*y*_2_	*γ*_*xxxx*_
[6]IF-CNB (*D*_3d_)	0.398	0.398	0.134	213
[8]IF-CNB (*D*_4_)	0.526	0.210	0.210	698
[8]IF-CNB^2+^ (*D*_4d_)	0.471	0.458	0.143	−2691

Similar to the case of [6]IF-CNB, we can suggest an analogy of the frontier MO levels of [8]IF-CNB to those of COT. Within the Hückel approximation, COT with BLA-less regular octagon (*D*_8h_) structure is known to have degenerate HOMO/LUMO levels. When the symmetry of COT reduces to *D*_4h_, the HOMO–LUMO gap is expected to increase owing to the structural distortion, whereas the degenerate level structure for other π-orbitals is considered to be preserved.^43^ The increase of HOMO–LUMO gap due to the reduction of symmetry is related to the decrease of *y*_0_ value. Actually, metastable structure of [8]IF-CNB with higher *C*_4v_ symmetry is found to have *y*_0_ = 1 (see Table S2†). Similar tendency was also discussed in the *D*_4h_ (*y*_0_ ∼ 1) and *D*_2h_ (*y*_0_ ∼ 0.1) structures of TCPTP,^28^ but in the present case, *y*_0_ value of the most stable local minimum structure of [8]IF-CNB with reduced *D*_4_ symmetry still exhibit intermediate value. This may originate in the structural features of [*N*]IF-CNB, namely, indeno[1,2-*b*]fluorene substructures are involved in the smoothly curved π-surface in the belt. When we remove two electrons from the HOMO of [8]IF-CNB, this MO becomes unoccupied level. In dicationic [8]IF-CNB^2+^, *y*_0_ and *y*_1_ values are found to be very close to each other, which is considered to reflect the degenerate HOMO/HOMO−1 and LUMO/LUMO+1 levels (see Fig. 6). The reason why *y*_0_ and *y*_1_ differ very slightly may be related to the theoretical and numerical treatments of evaluating *y*. In the case of [8]IF-CNB^2+^, *y*_0_ and *y*_1_ values (0.471 and 0.458, respectively) are found to be in the intermediate region.

**Fig. 7:**
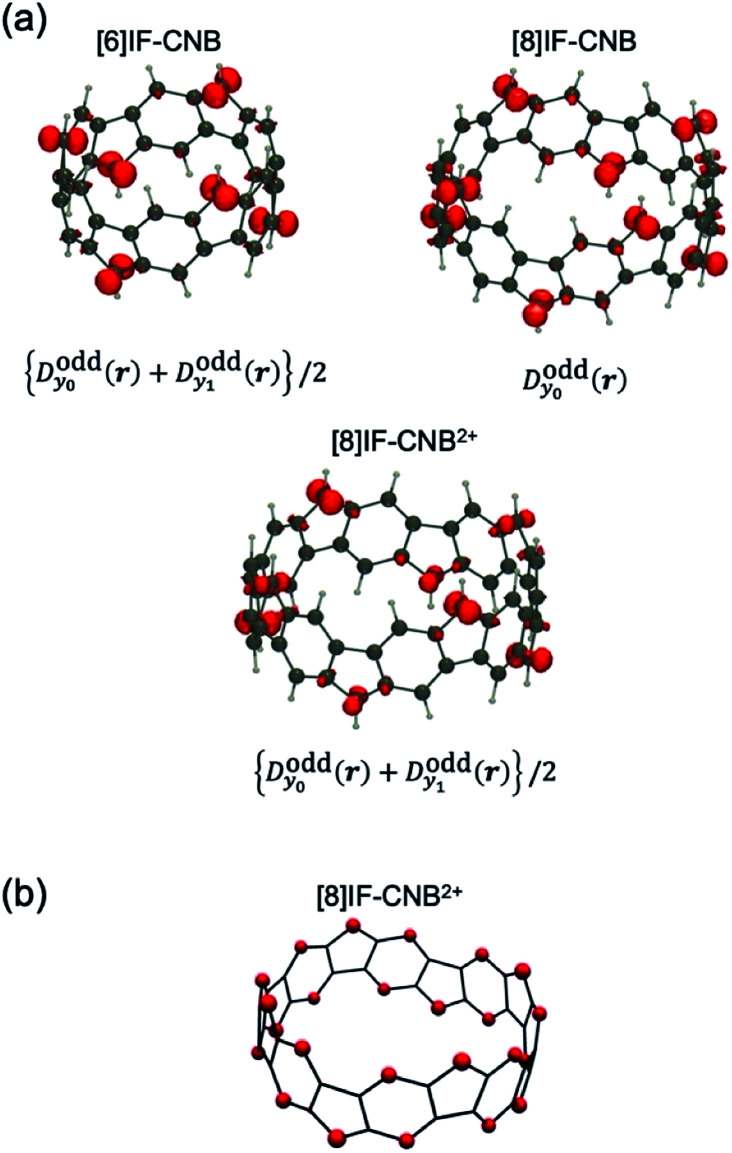
Odd-electron density distributions (a) and net Hirshfeld charges where the contributions from hydrogen atoms are summed into heavy atoms (b).

In order to investigate how and where the unpaired electrons distribute, we illustrate the maps of odd-electron densities for each system (see Fig. 7a). Note that we only plot the odd-electron densities from the contributions of *y*_0_ ([8]IF-CNB) or average of *y*_0_ and *y*_1_ ([6]IF-CNB and [8]IF-CNB^2+^). In all the systems, odd-electron densities are found to distribute primarily on the vertices of five-membered rings. Owing to the high symmetries of these systems (even for [8]IF-CNB), the amplitude of odd-electron densities on each vertex is found to be equivalent. In Fig. 7b, we also show the net Hirshfeld charge of [8]IF-CNB^2+^ in order to investigate the distributions of positive charges. In contrast to the odd-electron density maps, positive charges are found to be distributed on the six-membered rings as well as the vertices of five-membered rings. Such delocalized features of positive charges in [8]IF-CNB^2+^ may be reflected in the response properties of this system.

**Fig. 8:**
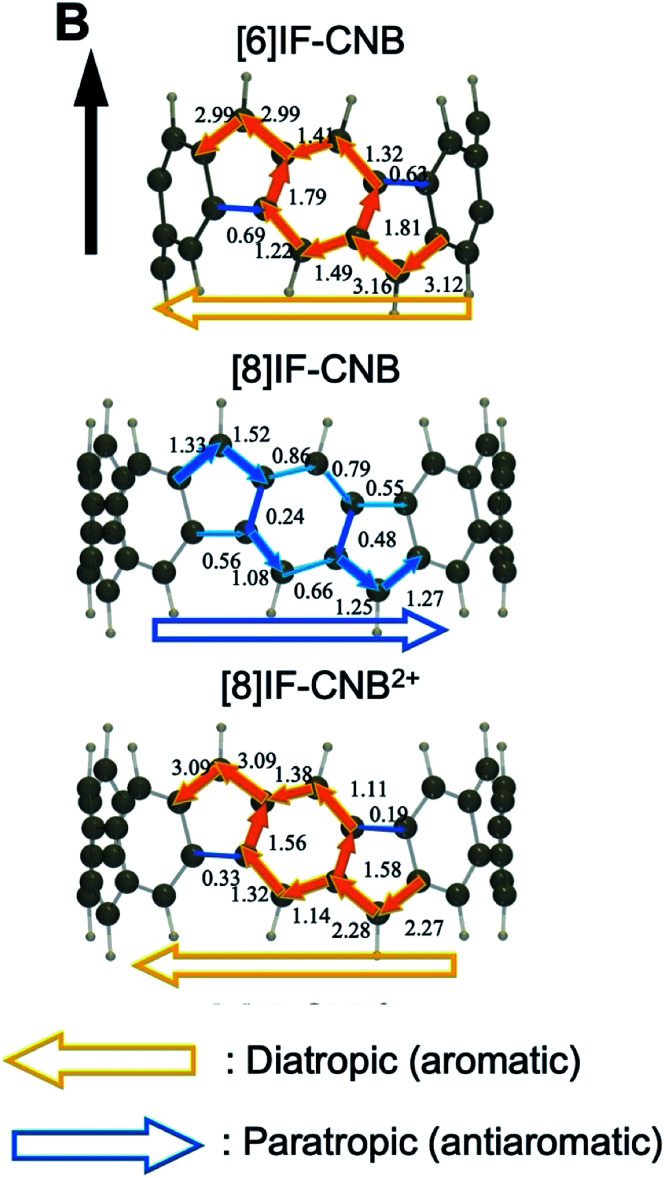
Maps of bond-integrated MIC densities. Orange left-arrows and blue right-arrows represent diatropic and paratropic contributions when the magnetic field *B* is applied along the present direction, respectively.

These unique geometric features and electronic structures of [*N*]IF-CNB are expected to be reflected in their aromatic characters. Among several criteria for evaluating aromatic characters, we here employed the GIMIC method, which is a powerful tool to qualitatively and (semi-)quantitatively understand the aromatic nature of conjugated systems. In our previous paper, we have found from the analysis of GIMIC that magnetically-induced current primarily originating in the π-electrons can flow over the belt along the curved π-surface, and as a result of the balance between the paratropic and diatropic contributions, [6]IF-CNB is expected to exhibit global aromatic nature, even though the amplitudes of MIC density are weak compared to those of benzene (see also Fig. 8).^29^Fig. 8 shows the side views of the maps of bond-integrated MIC densities for all the calculated systems. Note that, in Fig. 8, left- and right-arrows for the bond-integrated MIC density correspond to the diatropic (aromtic) and paratropic (antiaromatic) contributions in the induced ring currents, respectively. The values are the amplitudes of bond-integrated MIC density in the unit of nA T^−1^. Note that, the value of bond-integrated MIC density for benzene at the same calculation level is more than 10 nA T^−1^. From the maps, the main circuit of the flow of MIC is considered to be that along the repeating *p*QM substructure. In contrast to the results of [6]IF-CNB, paratropic contributions are found to become predominant in the ring current in [8]IF-CNB. Judging from the amplitudes of bond-integrated MIC densities (0.2–1.6 nA T^−1^) and the direction of flow, [8]IF-CNB is expected to exhibit weak global antiaromatic character. In the case of the dicationic [8]IF-CNB^2+^, directions and amplitudes of bond-integrated MIC densities are found to become inverted for bonds of *p*QM substructures, the feature of which suggests that [8]IF-CNB^2+^ is expected to exhibit weak global aromatic character like [6]IF-CNB. The tendencies of aromaticity analyzed by the GIMIC method are in consistent with those predicted from *n*_π_.

**Fig. 9:**
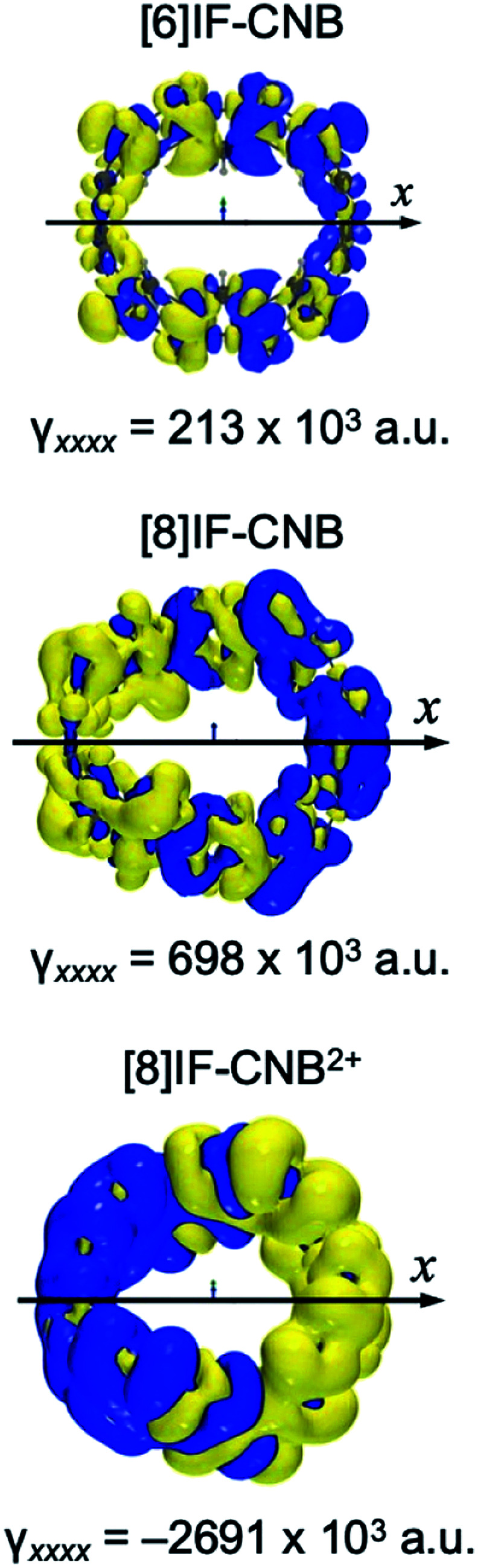
Maps of *γ*_*xxxx*_ densities. Yellow/blue meshes represent the isosurfaces of *γ*_*xxxx*_ density with the contour values of ±200 a.u., respectively.

The delocalized and intermediate open-shell characters of these systems suggest unique physico-chemical properties, such as, enhancement of third-order NLO properties. Actually, [6]IF-CNB was found to exhibit enhanced third-order NLO properties compared to its closed-shell counterpart [(6,6)CNB].^29^ In Table 1, we also summarized static *γ*_*xxxx*_ of these systems. In Fig. 9, we plot the *γ*_*xxxx*_ densities. The *γ*_*xxxx*_ value of [8]IF-CNB (698 × 10^3^ a.u.) is shown to be enhanced more than 300% from that of [6]IF-CNB (213 × 10^3^ a.u.). As seem from the *γ*_*xxxx*_ density maps which visualize the field-induced third-order electron polarization, polarizations of π-electrons on the curved π-surface are found to be the primary contributions of the total third-order response properties. This is considered to be related to the delocalized nature of π-electrons with intermediate open-shell characters. Drastic changes of *γ*_*xxxx*_ can be found in dicationic [8]IF-CNB^2+^ (−2691 × 10^3^ a.u.). The amplitude of *γ*_*xxxx*_ for [8]IF-CNB^2+^ is 3.86 times as large as that of [8]IF-CNB. Furthermore, the sign of *γ*_*xxxx*_ in [8]IF-CNB^2+^ is predicted to be negative at the present level of approximation, which can be visually illustrated by the inverted direction of third-order polarization in the *γ*_*xxxx*_ density map (see Fig. 9). Such a negative longitudinal *γ* value can sometimes be observed in charged soliton or charged radical species.^44–46^

In order to investigate the origins of negative *γ*_*xxxx*_ value of [8]IF-CNB^2+^, we evaluated the static polarizabilities (*α*_*xx*_) for these systems at the same level as that for *γ*_*xxxx*_ (see Table 1). On the basis of the sum-over-states expression for the symmetric systems within the three levels (0, *n* and *m*), the *γ*_*xxxx*_ value is approximately expressed as,^44^1
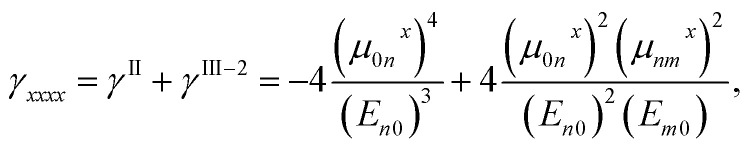
where *μ*_0*n*_^*x*^ and *E*_*n*0_ = *E*_*n*_ − *E*_0_ are the *x*-axis component of transition dipole moment between the states 0 (ground state) and *n* (excited state), and excitation energy for state *n*, respectively. Since excitation energy is positive, the first term *γ*^II^, which is called type-II term, contributes negatively to total *γ*_*xxxx*_ within the present approximation. In order to analyze the origin of negative *γ*_*xxxx*_, from the sum-over-states expression, qualitative and (semi-)quantitative excited state calculations are needed. Such a calculation for the present large-sized delocalized multiradicaloids and its charged species at the same accuracy demand significant computational effort. On the other hand, *γ*^II^ is related to *α*_*xx*_ as,^44^2
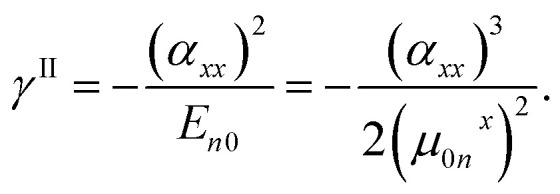


Therefore, even though the sign of full sum-over-states expression of *γ*_*xxxx*_ is determined by the balance of negative and positive terms (*γ*^II^ and *γ*^III−2^), the amplitude of *α*_*xx*_ may give information to understand the origin of negative *γ*_*xxxx*_.^44^ The *α*_*xx*_ value of [8]IF-CNB^2+^ (1600 a.u.) is found to be about 1.54 times as large as that of [8]IF-CNB (1040 a.u.) (1.54^2^ = 2.37). Large *α*_*xx*_ value is usually related to the small excitation energy for optically-allowed excited states and the large amplitudes of transition moments for these states. Even though there are several possible reasons, it is speculated that there is a possibility of existing such excited states in the dicationic [8]IF-CNB^2+^. Similar tendencies of reduction of excitation energy and negative *γ* were also predicted in the charged radical species like pentalene and *s*-indacene, the electronic structures of which are called symmetric resonance structures with invertible polarization (SRIP).^45,46^ Since we can illustrate several equivalent canonical forms in the resonance structures where radicals and positive charges appear on different sites, there may be a connection to the mechanism of SRIP. As another possible explanation, we may suggest interference effects of virtual excitation pathways appearing in *γ*^III−2^ of the full sum-over-states expression. Further analysis of excited states for such large-sized open-shell systems will be an interesting topic from the viewpoints of developing methods of quantum chemistry and controlling molecular functionalities, and thus studies toward this direction is still ongoing by using the multi-reference multi-state electron correlation methods.

## Conclusions

In this study, we have investigated the geometric features, electronic structures and third-order NLO properties of [6]IF-CNB, [8]IF-CNB and [8]IF-CNB^2+^, in order to clarify the relationship between *n*_π_, aromatic and open-shell characters, and physico-chemical properties of theoretically designed [*N*]IF-CNB. [*N*]IF-CNBs, which involve repeating indeno[1,2-*b*]fluorene substructures on the curved π-surface, are shown to have highly symmetric local minimum structures depending on *n*_π_. The most stable local minimum structures of [6]IF-CNB and [8]IF-CNB^2+^ with *n*_π_ = 4*n* + 2 are found to belong to highest possible symmetry with BLA-less nature of characteristic bonds. All the five- and six-membered rings in these systems are equivalent to each other, suggesting their delocalized electronic structures over the belt. In contrast, the most stable local minimum structure of [8]IF-CNB with *n*_π_ = 4*n* is shown to belong to reduced *D*_4_ symmetry. Finite alternation patterns for the specific CC bonds and for the distance between the vertices of adjacent five-membered rings suggest that there are quinoid- and benzenoid-like *p*QM substructures that appear alternately in the belt. Owing to the existence of *p*QM substructures, all these systems are expected to have intermediate open-shell characters. It is found that these systems have degenerate frontier MO levels depending on *n*_π_, the feature of which is reflected in (nearly) degenerate *y*_*i*_ values. Aromatic characters of [8]IF-CNB and its dicationic state evaluated by the GIMIC method are in consistent with those predicted from *n*_π_. Therefore, aromaticity classifications by *n*_π_ are expected to be applicable for [*N*]IF-CNB with larger *N* and their two-electron oxidized species. For the third-order NLO properties, two-electron oxidation of [8]IF-CNB is expected to enhance drastically the amplitude of *γ*_*xxxx*_ value, whereas the sign of *γ*_*xxxx*_ is inverted. We speculated that the negative value of *γ*_*xxxx*_ of [8]IF-CNB^2+^ is related to the enhancement of polarizability based on SRIP mechanism, which is associated with lowering of the excitation energies and raising of the transition moment amplitudes of the optically-allowed excited states, even though further analysis of excited states by using highly accurate quantum chemical calculations are needed in order to clarify the mechanisms.

Even though there are plenty of barriers to realize such fully conjugated open-shell macrocycles, such as ring-strain during the aromatization of the belt, requirements of bulky substituents at the vertices of five-membered rings to increase kinetic stabilities, *etc.*, the present theoretical results suggest that [*N*]IF-CNB and related materials are attractive targets of organic synthesis for realizing novel open-shell multi-functional conjugated macrocycles. For example, [*N*]IF-CNB may also be able to offer different electrostatic environments in inner and outer spatial domain, the feature of which can be useful for host–guest chemistry, like [*N*]CPPs.^12–17^ Very recently, synthesis of methylene-bridged [6]CPP has been reported by Segawa, Itami and coworkers.^47^ Furthermore, some well-designed open-shell conjugated molecules are known to form unique molecular aggregates where covalent-like intermolecular interactions exist. Since variation of *n*_π_ is expected to drastically change the structures and properties of [*N*]IF-CNB, electron transfer between host and guests, which may be triggered during the trapping of guests or by external stimuli, will be an interesting strategy for establishing novel dynamic open-shell functional materials and their control schemes. Studies toward such directions as well as further investigation on the size and charged-/spin-state dependences of the structures and properties of [*N*]IF-CNB and related systems are ongoing project in our group.

## Conflicts of interest

There are no conflicts to declare.

## Supplementary Material

RA-010-D0RA04787B-s001
