# Supplementary material for: Theoretical study on aromatic and open-shell characteristics of carbon nanobelts composed of indeno[1,2-b]fluorene units: dependence on the number of units and charge states
Source: RSC Adv. 2020 Jul 7;10(43):25736–45. doi: 10.1039/d0ra04787b (PMC9055345; doi:10.1039/d0ra04787b)
Supplement: RA-010-D0RA04787B-s001 [file RA-010-D0RA04787B-s001.pdf]

Electronic Supplementary Material (ESI) for RSC Advances.
This journal is © The Royal Society of Chemistry 2020

Electronic Supplementary Information

Theoretical study on aromatic and open-shell characters of carbon nanobelts composed of indeno[1,2-*b*]fluorene units: Dependence on the number of units and charge states

Ryohei Kishi,[a,b] Masaki Yamane,[a] Ryosuke Sugiura,[a] Wataru Yoshida,[a] Yosuke Shimizu[a] and Masayoshi Nakano[a, b, c*]

a. Department of Materials Engineering Science, Graduate School of Engineering Science, Osaka University, 1-3 Machikaneyama, Toyonaka, Osaka 560-8531, Japan. Email: mnaka@cheng.es.osaka-u.ac.jp

b. Center for Quantum Information and Quantum Biology (QIQB), Institute for Open and Transdisciplinary Research Initiatives, Osaka University, 1-3 Machikaneyama, Toyonaka, Osaka 560-8531, Japan.

c. Center for Spintronics Research Network (CSRN), Graduate School of Engineering Science, Osaka University, 1-3 Machikaneyama, Toyonaka, Osaka 560-8531, Japan.

Contents

1. **Possible resonance structures of [8]IF-CNB.**
2. **Dependence of the optimized geometries on the calculation method.**
3. **Metastable local minimum structure of [8]IF-CNB with C_{4v} symmetry.**
4. **Cartesian coordinates of each system.**
5. **References.**

1. Possible resonance structures of [8]IF-CNB.

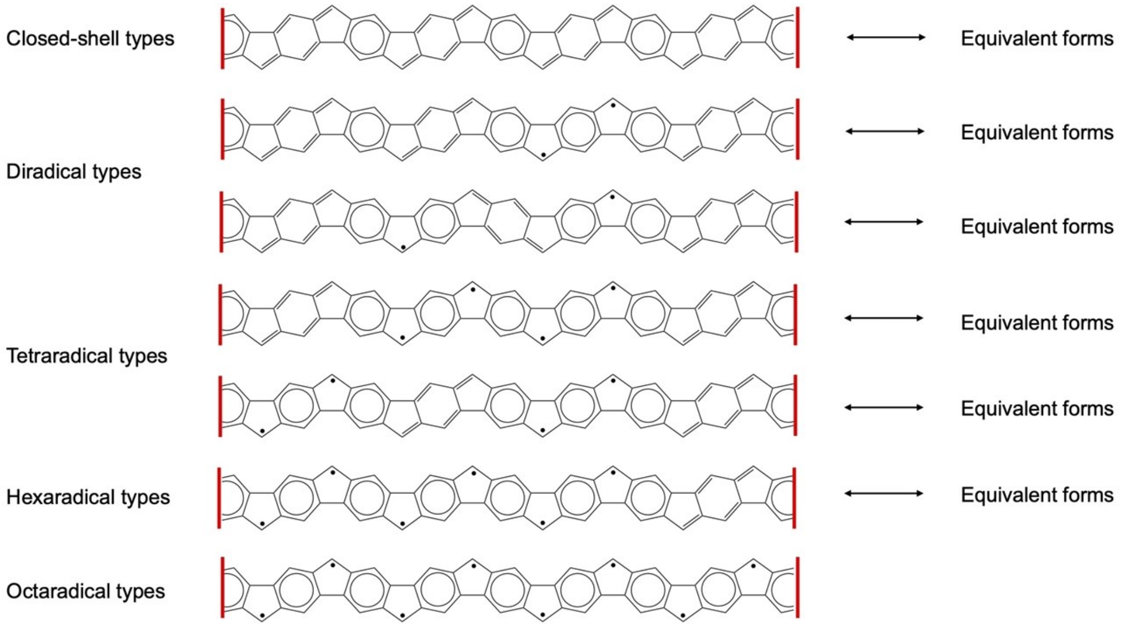

Figure S1. Possible resonance forms of [8]IF-CNB. Both-ends red lines represent the cutting line of the hoop.

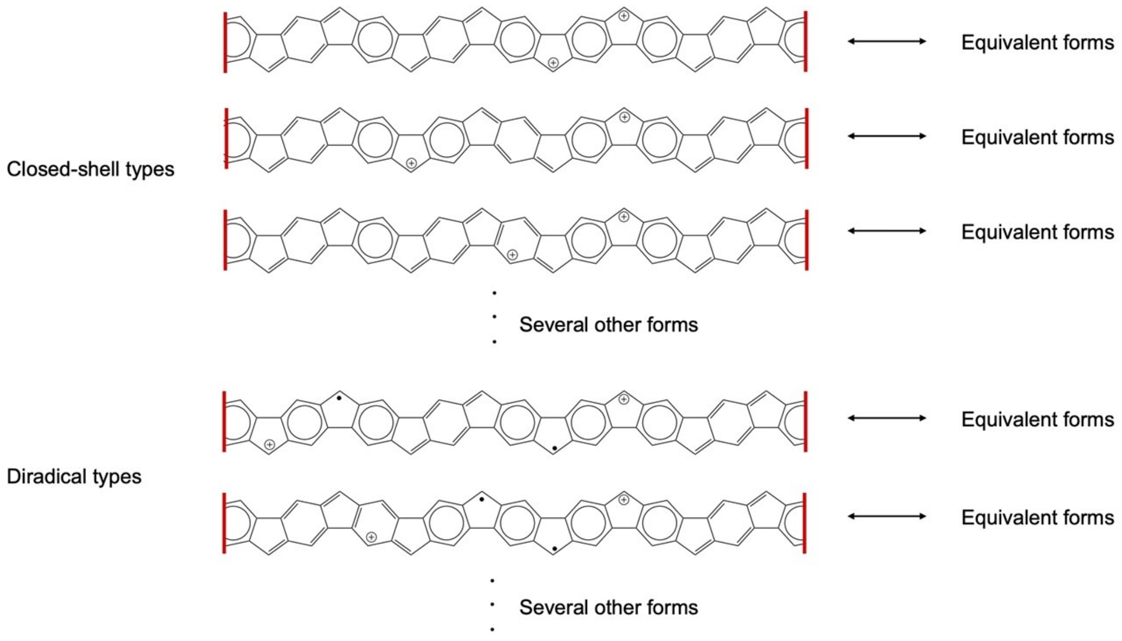

Figure S2. Examples of possible resonance forms of [8]IF-CNB^{2+} (up to diradical types). Both-ends red lines represent the cutting line of the hoop.

2. Dependence of the optimized geometries on the calculation method.

Exploring appropriate choice of theoretical method for geometry optimizations of intermediate di- and multi-radicaloids with extended π-conjugations is one of the most important topics in the chemistry of open-shell molecules. A given approximate method will sometimes predict totally different geometric features and resulting physico-chemical properties depending on how electron correlation effect is treated. In our previous studies[1-3], we have carefully compared the optimized geometries for linear oligomers of condensed-ring conjugated systems obtained at the spin-restricted (R)B3LYP and unrestricted (U)B3LYP levels with those at the spin-flip time-dependent density functional theory (SF-TDDFT) or with experimental geometries. From the calculation results of intermediate multiradicaloids composed of five- and six-membered rings, UB3LYP tends to predict unreasonably elongated bond lengths around which unpaired electrons are distributing. This is considered to be primarily due to the spin-contamination effects in UDFT method. On the other hand, it was found that RB3LYP is expected to reproduce better the bond-length alternation (BLA) patterns of intermediate multi-radicaloids if diradical characters (y_i) are not so high. Overall, geometry optimizations using RB3LYP exchange-correlation functional along with valence triple-zeta basis set (such as 6-311G*) have been shown to predict appropriate geometric features of several finite-size open-shell multi-radicaloids.

On the other hand, from the viewpoint of cyclic structures, π-conjugation lengths of the present conjugated macrocycles are regarded as infinite, and thus, their electronic structures are closely related to those of one-dimensional polymers, where the description of electron correlation is also considered to be important for the predictions of geometries and properties. Indeed, BLA patterns of fully π-conjugated macrocycles and of one-dimensional polymers have also been studied actively.

From these viewpoints, we have investigated dependence of optimized geometries on the calculation method. At first, we compared the results of spin-restricted DFT methods, namely, global hybrid RB3LYP (Hartree-Fock exchange: 20%), RPBE0 (25%), RM06-2X (54%), range-separating hybrid RCAM-B3LYP, as well as RHF for neural [6]IF-CNB (see Fig. S3).

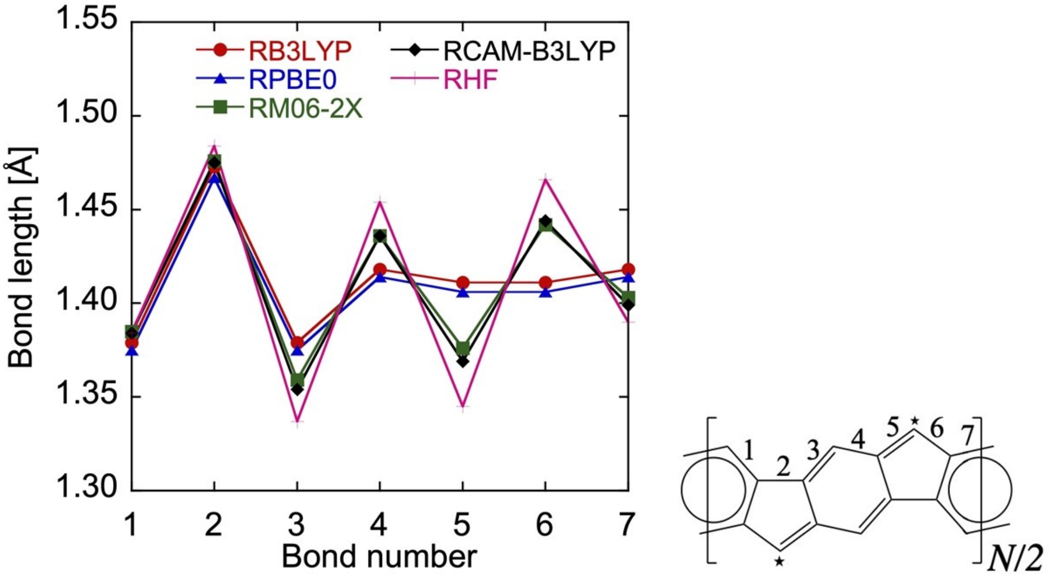

Figure S3. Comparison of bond-lengths of selected CC bond for [6]IF-CNB by RDFT methods.

As is explained in the main text of the manuscript, RB3LYP is found to predict a local minimum structure where CC bonds around the vertex of five-membered ring (bonds 5 and 6) are BLA-less, meaning that two canonical forms in Fig. 3a contribute equivalently in the resonance structure. RPBE0 also predicted a BLA-less local minimum structure, namely, RB3LYP and RPBE0 tend to predict the delocalized electronic structures. On the other hand, such a BLA-less structure is predicted to be saddle-point structures on the potential energy surface (PES) at the RHF, RM06-2X and RCAM-B3LYP levels. At these levels of approximation, we found a finite BLA around the bonds 5 and 6, meaning that one of the canonical forms in Fig. 3a is considered to contribute primarily in the resonance structure in [6]IF-CNB. These methods tend to prefer bond-localized electronic structures. The optimized structures at these different DFT methods can be classified into two types; for simplicity, we call them alternated (RHF, RM06-2X and RCAM-B3LYP) and less-alternated (RB3LYP and PBE0) types, respectively.

Such a calculation method dependence of the optimized geometries is considered to be related to the amount of HF exchange involved in the hybrid DFT methods. For π-conjugated systems, HF method tends to predict the bond-localized electronic structure in general, whereas the (pure) DFT method does delocalized one. When the amount of HF

exchange becomes large, bond-localized structure becomes relatively more stable than delocalized one.

Such a localized/delocalized nature of electronic structure is also related to their open-shell characters. Unfortunately, HOMO and HOMO-1 (LUMO and LUMO+1) of [6]IF-CNB are predicted to degenerate with each other, and thus, SF-TDDFT method starting from the $Ms = 1$ triplet state may be insufficient to describe the electronic structure of this system. Thus, we have performed geometry optimizations at the CASSCF(4e,4o) level, starting from both the alternated and less-alternated initial geometries. On the other hand, description of dynamical correlation is also important to characterize the localized/delocalized nature of electrons. In order to examine the effect of dynamical correlation on the total energies, we have performed additional single point calculations using the CASSCF optimized geometries at the strongly-contracted *n*-electron valence state perturbation theory (SC-NEVPT2) level. [4] These multi-reference calculations were performed using ORCA 4.2 program package. [5] In order to reduce the computational efforts, resolution of the identity (RI) approximation for the integral evaluations was employed during the CASSCF and SC-NEVPT2 calculations, where the automatic generation procedure for auxiliary basis functions implemented in ORCA (with the keyword “AutoAux”) was employed. No symmetry constraint was imposed during the calculations. We have numerically constructed the Hessian matrix for each local minimum in order to perform CASSCF frequency analysis.

Table S1 shows the summary of calculation results at the CASSCF and SC-NEVPT2 levels. We obtained both alternated and less-alternated stationary point structures from the CASSCF geometry optimizations. The CASSCF total energy of alternated structure is found to be lower than that of less-alternated structure, and from the results of frequency analysis, alternated and less-alternated structures are predicted to be a local minimum and saddle-point structures, respectively. Since y_0/y_1 values at the CASSCF level are found to be smaller than 0.1, such a BLA features of CASSCF geometry at the local minimum is similar to that of single reference RHF method. Actually, CASSCF local minimum geometry is found to be very similar to RHF one (see Fig. S2). However, when we evaluated the single point SC-NEVPT2 energies on these stationary point structures, the order of the total energy becomes inverted, namely, less-alternated structure is energetically more stable than the alternated one. Even though the present single point

SC-NEVPT2 calculations may not be sufficient enough to describe the electronic structures of these systems, these results suggest that the description of dynamical correlation is very important in the present case.

Table S1. Summary of calculation results at the CASSCF and SC-NEVPT2 levels.

Structure type	Alternated	Less-alternated
# of imaginary frequency	0 (local minimum)	1 (saddle-point)
CASSCF total energy [a.u.]	–1600.96308	–1600.94923
y_0/y_1 at the CASSCF level [-]	0.0250/0.0250	0.0905/0.0905
Single point SC-NEVPT2 energy [a.u.]	–1606.55218	–1606.59746

In general, geometry optimization at the multi reference PT2 level demands huge computational effort. Since y_0/y_1 values at the CASSCF level are not so high, we alternately have performed the single reference RMP2 geometry optimizations for this system, in order to clarify which type of structure is the local minimum of [6]IF-CNB. Note that, RMP2 method was reported to be useful for the geometry optimizations of oligoacenes with small-intermediate open-shell characters[6]. Again, RI approximation was employed during the RMP2 calculations using ORCA program package.

Calculation results are shown in Fig. S4. Unfortunately, RMP2 Hessian calculation for this system is found to demand high computational effort, and thus we could not have obtained the results of frequency analysis at the RMP2 level. However, we should note that we could not have obtained a stationary point with alternated structure, even though we started from both the alternated initial geometries. From these results, RMP2 is considered to predict the less-alternated structure. Furthermore, the BLA features of RMP2 geometry are considered to be reproduced well by the RB3LYP method, although slight deviations of bond-lengths (~ 0.01 Å at the maximum) exist.

Judging from the calculation method dependence of the optimized geometry discussed here, we have employed the RB3LYP method for the geometry optimizations of [*N*]IF-CNBs studied here. Of course, when *N* is increasing, the electronic structure of [*N*]IF-CNB is considered to become closer to its one-dimensional counterpart (polymer). In such case, whether less-alternated structure still becomes a local minimum or not should

be carefully discussed by comparing results at several possible approximation methods.

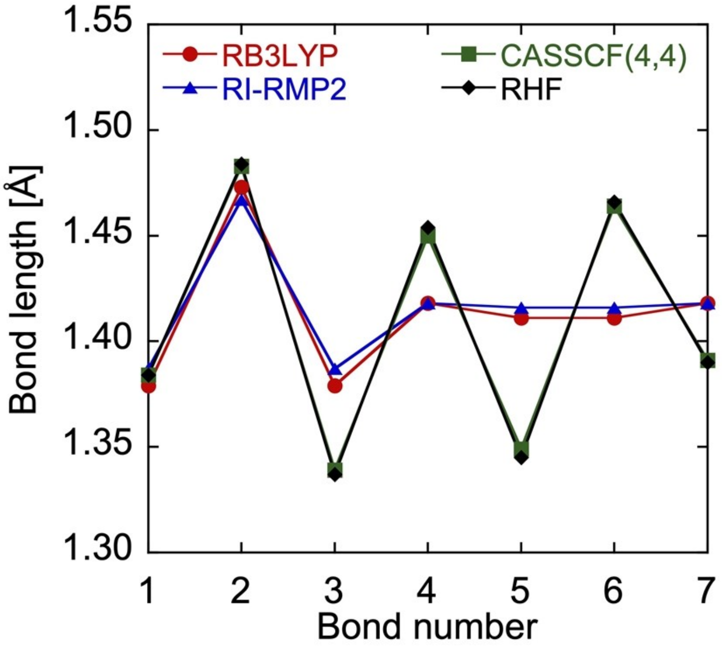

Figure S4. Comparison of bond-lengths of selected CC bond for [6]IF-CNB by *ab initio* correlation methods.

3. Metastable local minimum structure of [8]IF-CNB with C_{4v} symmetry.

Table S2. Summary of calculation results of diradical characters y_i [-] and γ_{xxxx} [10^3 a.u.] for the obtained local minimum structures.

System	y_0	y_1	y_2	γ_{xxxx}
[6]IF-CNB (D_{3d})	0.398	0.398	0.134	213
[8]IF-CNB (C_{4v})	1.000	0.271	0.271	816
[8]IF-CNB (D_4)	0.526	0.210	0.210	698
[8]IF-CNB^{2+} (D_{4d})	0.471	0.458	0.143	−2691

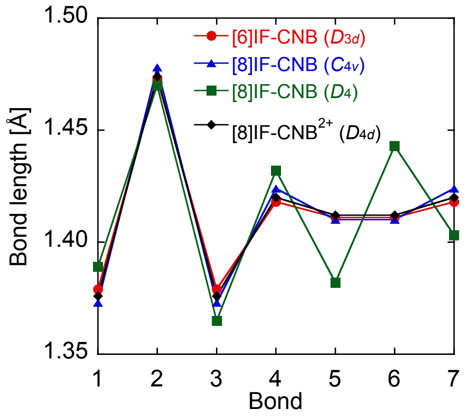

Figure S5. Bond lengths of CC bonds of [8]IF-CNB with C_{4v} symmetry.

4. Cartesian coordinates of each system.

Table S3. Cartesian coordinates [Å] of the optimized structure of [8]IF-CNB (D_4).

Atom	x	y	z
C	-3.422084	4.077153	1.367499
C	-2.710411	4.647475	0.351992
C	-3.057320	4.374361	-1.044030
C	-4.077153	3.422084	-1.367499
C	-4.647475	2.710411	-0.351992
C	-4.374361	3.057320	1.044030
C	-0.937800	5.268298	-1.026179
C	-1.356520	5.220378	0.342415
C	-5.268298	0.937800	1.026179
C	-5.220378	1.356520	-0.342415
C	0.422916	5.313100	-1.366815
C	1.356520	5.220378	-0.342415
C	-0.422916	5.313100	1.366815
C	0.937800	5.268298	1.026179
C	-5.313100	-0.422916	1.366815
C	-5.220378	-1.356520	0.342415
C	-5.313100	0.422916	-1.366815
C	-5.268298	-0.937800	-1.026179
C	-3.057320	-4.374361	1.044030
C	-2.710411	-4.647475	-0.351992
C	-4.374361	-3.057320	-1.044030
C	-4.647475	-2.710411	0.351992
C	-4.077153	-3.422084	1.367499
C	-3.422084	-4.077153	-1.367499
C	-0.937800	-5.268298	1.026179
C	-1.356520	-5.220378	-0.342415
C	0.422916	-5.313100	1.366815
C	-0.422916	-5.313100	-1.366815

C	1.356520	-5.220378	0.342415
C	0.937800	-5.268298	-1.026179
C	2.710411	-4.647475	0.351992
C	3.422084	-4.077153	1.367499
C	3.057320	-4.374361	-1.044030
C	4.374361	-3.057320	1.044030
C	4.077153	-3.422084	-1.367499
C	4.647475	-2.710411	-0.351992
C	5.220378	-1.356520	-0.342415
C	5.268298	-0.937800	1.026179
C	5.313100	-0.422916	-1.366815
C	5.313100	0.422916	1.366815
C	5.268298	0.937800	-1.026179
C	5.220378	1.356520	0.342415
C	4.647475	2.710411	0.351992
C	4.374361	3.057320	-1.044030
C	4.077153	3.422084	1.367499
C	3.422084	4.077153	-1.367499
C	3.057320	4.374361	1.044030
C	2.710411	4.647475	-0.351992
C	-2.038944	4.838783	-1.854047
C	-4.838783	2.038944	1.854047
C	-4.838783	-2.038944	-1.854047
C	-2.038944	-4.838783	1.854047
C	2.038944	-4.838783	-1.854047
C	4.838783	-2.038944	1.854047
C	4.838783	2.038944	-1.854047
C	2.038944	4.838783	1.854047
H	-3.151905	4.236020	2.408035
H	-4.236020	3.151905	-2.408035
H	0.723062	5.229760	-2.407709
H	-0.723062	5.229760	2.407709

H	-5.229760	-0.723062	2.407709
H	-5.229760	0.723062	-2.407709
H	-4.236020	-3.151905	2.408035
H	-3.151905	-4.236020	-2.408035
H	0.723062	-5.229760	2.407709
H	-0.723062	-5.229760	-2.407709
H	3.151905	-4.236020	2.408035
H	4.236020	-3.151905	-2.408035
H	5.229760	-0.723062	-2.407709
H	5.229760	0.723062	2.407709
H	4.236020	3.151905	2.408035
H	3.151905	4.236020	-2.408035
H	-4.736497	1.994834	2.932227
H	-4.736497	-1.994834	-2.932227
H	-1.994834	-4.736497	2.932227
H	1.994834	-4.736497	-2.932227
H	4.736497	-1.994834	2.932227
H	4.736497	1.994834	-2.932227
H	1.994834	4.736497	2.932227
H	-1.994834	4.736497	-2.932227

Table S4. Cartesian coordinates [Å] of the optimized structure of [8]IF-CNB^{2+}(D_{4d}).

Atom	x	y	z
C	5.314040	-0.437945	-1.372768
C	5.209575	-1.360302	-0.357092
C	5.262892	-0.936348	1.020208
C	5.314061	0.437924	1.372768
C	5.209871	1.360318	0.357074
C	5.263146	0.936354	-1.020209
H	5.262439	-0.727841	-2.417523
H	5.262377	0.727771	2.417537
C	4.383463	-3.059417	1.020208

C	4.645550	-2.721941	-0.357092
C	4.383771	3.059416	-1.020209
C	4.645881	2.721951	0.357074
C	3.447868	-4.067337	1.372768
C	2.721951	-4.645881	0.357074
H	3.206365	-4.235739	2.417537
C	4.067198	-3.448002	-1.372768
C	3.059416	-4.383771	-1.020209
H	4.235702	-3.206531	-2.417523
C	3.448002	4.067198	-1.372768
C	2.721941	4.645550	-0.357092
H	3.206531	4.235702	-2.417523
C	4.067337	3.447868	1.372768
C	3.059417	4.383463	1.020208
H	4.235739	3.206365	2.417537
C	-0.936354	5.263146	-1.020209
C	-1.360318	5.209871	0.357074
C	0.936348	5.262892	1.020208
C	1.360302	5.209575	-0.357092
C	0.437945	5.314040	-1.372768
H	0.727841	5.262439	-2.417523
C	-0.437924	5.314061	1.372768
H	-0.727771	5.262377	2.417537
C	-3.059416	4.383771	-1.020209
C	-2.721951	4.645881	0.357074
C	-4.067198	3.448002	-1.372768
C	-3.447868	4.067337	1.372768
C	-4.645550	2.721941	-0.357092
H	-4.235702	3.206531	-2.417523
C	-4.383463	3.059417	1.020208
H	-3.206365	4.235739	2.417537
C	-5.209575	1.360302	-0.357092

C	-5.314040	0.437945	-1.372768
C	-5.262892	0.936348	1.020208
C	-5.263146	-0.936354	-1.020209
H	-5.262439	0.727841	-2.417523
C	-5.314061	-0.437924	1.372768
C	-5.209871	-1.360318	0.357074
H	-5.262377	-0.727771	2.417537
C	-4.645881	-2.721951	0.357074
C	-4.383771	-3.059416	-1.020209
C	-4.067337	-3.447868	1.372768
C	-3.448002	-4.067198	-1.372768
C	-3.059417	-4.383463	1.020208
H	-4.235739	-3.206365	2.417537
C	-2.721941	-4.645550	-0.357092
H	-3.206531	-4.235702	-2.417523
C	-1.360302	-5.209575	-0.357092
C	-0.936348	-5.262892	1.020208
C	-0.437945	-5.314040	-1.372768
C	0.437924	-5.314061	1.372768
C	0.936354	-5.263146	-1.020209
H	-0.727841	-5.262439	-2.417523
C	1.360318	-5.209871	0.357074
H	0.727771	-5.262377	2.417537
C	4.858718	-2.012604	1.839310
C	4.859144	2.012666	-1.839310
C	2.012604	4.858718	1.839310
C	-2.012666	4.859144	-1.839310
C	-4.858718	2.012604	1.839310
C	-4.859144	-2.012666	-1.839310
C	-2.012604	-4.858718	1.839310
C	2.012666	-4.859144	-1.839310
H	4.789309	1.983740	-2.920154

H	1.983573	4.788631	2.920132
H	-1.983740	4.789309	-2.920154
H	-4.788631	1.983573	2.920132
H	-4.789309	-1.983740	-2.920154
H	-1.983573	-4.788631	2.920132
H	1.983740	-4.789309	-2.920154
H	4.788631	-1.983573	2.920132

Table S5. Cartesian coordinates [Å] of the optimized structure of [8]IF-CNB (C_{4v}; metastable structure).

Atom	x	y	z
C	-2.43234205	4.72531986	-1.36969995
C	-3.25541997	4.28790522	-0.34908599
C	-2.88148403	4.49988079	1.03340197
C	-1.62421298	5.07932711	1.36529696
C	-0.73884702	5.33787680	0.34878200
C	-1.15326095	5.21811819	-1.03412795
H	-2.67785501	4.53567219	-2.41135812
H	-1.31526697	5.11791801	2.40619111
C	-4.49988079	2.88148403	1.03340197
C	-4.28790522	3.25541997	-0.34908599
C	1.15326095	5.21811819	-1.03412795
C	0.73884702	5.33787680	0.34878200
C	-5.07932711	1.62421298	1.36529696
C	-5.33787680	0.73884702	0.34878200
H	-5.11791801	1.31526697	2.40619111
C	-4.72531986	2.43234205	-1.36969995
C	-5.21811819	1.15326095	-1.03412795
H	-4.53567219	2.67785501	-2.41135812
C	2.43234205	4.72531986	-1.36969995
C	3.25541997	4.28790522	-0.34908599

H	2.67785501	4.53567219	-2.41135812
C	1.62421298	5.07932711	1.36529696
C	2.88148403	4.49988079	1.03340197
H	1.31526697	5.11791801	2.40619111
C	5.21811819	1.15326095	-1.03412795
C	5.33787680	0.73884702	0.34878200
C	4.49988079	2.88148403	1.03340197
C	4.28790522	3.25541997	-0.34908599
C	4.72531986	2.43234205	-1.36969995
H	4.53567219	2.67785501	-2.41135812
C	5.07932711	1.62421298	1.36529696
H	5.11791801	1.31526697	2.40619111
C	5.21811819	-1.15326095	-1.03412795
C	5.33787680	-0.73884702	0.34878200
C	4.72531986	-2.43234205	-1.36969995
C	5.07932711	-1.62421298	1.36529696
C	4.28790522	-3.25541997	-0.34908599
H	4.53567219	-2.67785501	-2.41135812
C	4.49988079	-2.88148403	1.03340197
H	5.11791801	-1.31526697	2.40619111
C	3.25541997	-4.28790522	-0.34908599
C	2.43234205	-4.72531986	-1.36969995
C	2.88148403	-4.49988079	1.03340197
C	1.15326095	-5.21811819	-1.03412795
H	2.67785501	-4.53567219	-2.41135812
C	1.62421298	-5.07932711	1.36529696
C	0.73884702	-5.33787680	0.34878200
H	1.31526697	-5.11791801	2.40619111
C	-0.73884702	-5.33787680	0.34878200
C	-1.15326095	-5.21811819	-1.03412795
C	-1.62421298	-5.07932711	1.36529696
C	-2.43234205	-4.72531986	-1.36969995

C	-2.88148403	-4.49988079	1.03340197
H	-1.31526697	-5.11791801	2.40619111
C	-3.25541997	-4.28790522	-0.34908599
H	-2.67785501	-4.53567219	-2.41135812
C	-4.28790522	-3.25541997	-0.34908599
C	-4.49988079	-2.88148403	1.03340197
C	-4.72531986	-2.43234205	-1.36969995
C	-5.07932711	-1.62421298	1.36529696
C	-5.21811819	-1.15326095	-1.03412795
H	-4.53567219	-2.67785501	-2.41135812
C	-5.33787680	-0.73884702	0.34878200
H	-5.11791801	-1.31526697	2.40619111
C	-3.72193599	3.72193599	1.85663199
C	0.00000000	5.23496294	-1.84565794
C	3.72193599	3.72193599	1.85663199
C	5.23496294	0.00000000	-1.84565794
C	3.72193599	-3.72193599	1.85663199
C	0.00000000	-5.23496294	-1.84565794
C	-3.72193599	-3.72193599	1.85663199
C	-5.23496294	0.00000000	-1.84565794
H	0.00000000	5.11911201	-2.92421889
H	3.64186502	3.64186502	2.93390393
H	5.11911201	0.00000000	-2.92421889
H	3.64186502	-3.64186502	2.93390393
H	0.00000000	-5.11911201	-2.92421889
H	-3.64186502	-3.64186502	2.93390393
H	-5.11911201	0.00000000	-2.92421889
H	-3.64186502	3.64186502	2.93390393

5. References

[1] K. Fukuda, T. Nagami, J. Fujiyoshi, M. Nakano, *J. Phys. Chem. A* 2015, **119**, 10620.

[2] K. Fukuda, J. Fujiyoshi, H. Matsui, T. Nagami, S. Takamuku, Y. Kitagawa, B. Champagne, M. Nakano, *Org. Chem. Front.* 2017, **4**, 779.

[3] S. Nobusue, H. Miyoshi, A. Shimizu, I. Hisaki, K. Fukuda, M. Nakano, Y. Tobe, *Angew. Chem. Int. Ed.* 2015, **54**, 2090.

[4] F. Neese, *Wiley Interdisciplinary Reviews: Computational Molecular Science* 2012, Vol. 2, Issue 1, Pages 73-78; *ibid*, 2017, Vol. 8, Issue 1, p. e1327.

[5] C. Angeli, R. Cimiraglia, S. Evangelisti, T. Leininger, J.-P. Malrieu, *J. Chem. Phys.* 2001, **114**, 10252; C. Angeli, R. Cimiraglia, J.-P. Malrieu, *Chem. Phys. Lett.* 2001, **350**, 297; C. Angeli, R. Cimiraglia, J.-P. Malrieu, *J. Chem. Phys.* 2002, **117**, 9138.

[6] M. Pinheiro Jr., L. F. A. Ferrão, F. Bettanin, A. J. A. Aquino, F. B. C. Machado, H. Lischka, *Phys. Chem. Chem. Phys.* 2017, **19**, 19225.
